# Varicella Vaccine: a Molecular Variant That May Contribute to Attenuation

**DOI:** 10.1128/mbio.03120-22

**Published:** 2022-12-05

**Authors:** Anne A. Gershon, Michael D. Gershon

**Affiliations:** a Department of Pediatrics, Vagelos College of Physicians and Surgeons, Columbia University, New York, New York, USA; b Department of Pathology and Cell Biology, Vagelos College of Physicians and Surgeons, Columbia University, New York, New York, USA

**Keywords:** attenuation, vaccinology, varicella vaccines, varicella-zoster virus, virology

## Abstract

Varicella was troublesome when varicella vaccine (vOka) was licensed in the United States. Varicella’s yearly death toll was ~100, indirect costs were massive, and varicella threatened immunocompromised children. Since licensure, varicella has almost disappeared; nevertheless, vOka attenuation has lacked a molecular explanation. Sadaoka et al. (T. Sadaoka, D. P. Depledge, L. Rajbhandari, J. Breuer, et al., mBio 13:e0186422, 2022, https://doi.org/10.1128/mbio.01864-22), however, have now identified 6 core single nucleotide polymorphisms (SNPs), which singly or in combination may contribute to VOka attenuation; moreover, they found a predominant variant allele of vOka encoding the viral glycoprotein gB that results in glutamine instead of arginine at amino acid 699. This change impairs fusion activity and the ability of varicella-zoster virus (VZV) to infect human neurons from axon terminals. Molecular virological studies of vOka are reassuring in suggesting that reversion to virulence is unlikely and should also help assuage current fears about VZV vaccination and alleviate unanticipated future problems. The impressive work of Sadaoka et al. thus represents an auspicious advance in knowledge.

## COMMENTARY

In 1974, the year that Michiaki Takahashi published his successful attenuation of varicella-zoster virus (VZV), now called vOka (1), the number of yearly cases of varicella in the United States, ~4,000,000, was approximately equal to the birth cohort (National Center for Immunization and Respiratory Diseases, Division of Viral Diseases, Centers for Disease Control and Prevention, https://www.cdc.gov/chickenpox/vaccine-infographic.html#text). Every year, about 100 people died of the disease and 10,000 were hospitalized. Beyond these numbers, countless hours of school were missed, parents, who were forced to care for sick children, lost work, and virulent pathogens, including streptococci, were scratched into itching varicella-infected skin (2). In March 1995, after a great deal of Sturm und Drang, the varicella vaccine, containing vOka, was licensed in the United States. Since then, the National Institutes of Health (NIH) estimate that the varicella caseload has been reduced by 92%, varicella deaths by 90%, and varicella-related hospitalizations by 82% (https://www.cdc.gov/chickenpox/vaccine-infographic.html#text). The extraordinary success of varicella vaccine has been emblazoned in popular media (J. Winter, https://www.newyorker.com/science/elements/what-happened-after-the-chicken-pox-vaccine), and the lack of understanding of its attenuation has not interfered with the use of vOka. Now, in a recent issue of *mBio*, Sadaoka et al. have published a remarkable study that presages insight into the molecular changes that help to explain the attenuation of vOka (3). Essentially, the data identify that there are six core single nucleotide polymorphisms (SNPs) in vOka preparations, which may, singly, or in combination, be necessary or sufficient for vOka attenuation. Sadaoka et al. also found that vOka contains a predominant variant allele in ORF31, which encodes the viral glycoprotein gB (resulting in a glutamine at amino acid 699 instead of arginine), that impairs virus-induced fusion activity and the ability of virus to infect human neurons from axon terminals. This variant allele of ORF31 may thus contribute to the attenuation of vOka. Importantly, the work of Sadaoka et al., and others, suggests that the attenuation of vOka does not depend on a single allele, such as that identified in ORF31, which is reassuring because it means that reversion to virulence is highly unlikely.

It is impressive that the use of vOka to prevent varicella was finally licensed in the United States despite doubts, expressed on the front page of the *New York Times* (4), about how long vaccine-induced immunity to VZV infection would last and whether varicella was more of a nuisance than a threat to public health. These doubts stemmed from a fear that the prevention of the relatively mild childhood varicella could lead, after immunity waned, to varicella in adults, a much more dangerous disease. Another fear was that VZV causes latent infection and can reactivate. Reactivation of vOKa might thus lead to yet another infection, vOka-induced zoster. Worse yet, because no animal model of VZV infection was available, testing had to be performed on humans. By the time of licensure, however, vaccine-induced immunity had been demonstrated not to wane (5), an epidemic of vOka-induced zoster had failed to materialize, and sufficient testing on human subjects, which was greatly facilitated by the development of an immune correlate of immunity, the fluorescent antibody to membrane antigen (FAMA) test (6), had made the safety of varicella vaccination apparent (7). Human testing, which is the only definitive way to establish viral attenuation, had done so for vOka.

Importantly, at the time of varicella vaccine licensure, it had become evident that varicella was more than a nuisance of childhood. Successful treatment of leukemia and other disorders with medications that had the unwanted property of immunosuppression had come into wide use. Immunosuppression greatly enhances the severity of varicella, which therefore is a threat to the survival of immunosuppressed individuals. Children were thus being cured of leukemia but dying of varicella. Studies with the FAMA assay suggested that leukemic vaccinees would be protected if exposed to varicella. A subsequent NIH-supported collaborative investigation, involving about 500 leukemic children, revealed that 85% were completely protected against varicella following exposure to a varicella-infected sibling; none required antiviral therapy, and no severe cases of varicella occurred (7). This severe test of vOka in immunocompromised leukemic subjects enabled the vaccine to be evaluated and demonstrated to be safe and effective in the general population (8).

After the licensure of the varicella vaccine in the United States led to the virtual disappearance of varicella, the very success of the vaccine produced a new fear that has led to vaccination resistance in the United Kingdom and other countries. This fear, based on computer models, was that maintenance of immunity in adults required exposure to children with varicella (9). The absence of such children due to vaccine-induced childhood immunity would, it was predicted, cause adult immunity to wane and lead to the emergence of epidemic zoster. The predicted epidemic of zoster, however, has not occurred in the United States, despite its long experience with varicella vaccination (10). Sporadic outbreaks of varicella continued to occur following vaccine licensure, but their occurrence has now been controlled by introducing a second dose of vaccine. The failure of epidemic zoster to materialize implies that children do not have to be forced to endure varicella to protect adults. Decades ago, Hope-Simpson postulated that varicella-acquired VZV would periodically reactivate but would do so with a severity that was mild enough for the body to suppress the reactivated virus and keep the infection below a threshold necessary for manifestation of clinical disease (11). The postulated reactivations, however, would provide an endogenous boost to immunity. Zoster would appear when the force of endogenous boosting, as a function of age or other factors, became inadequate to maintain sufficient immunity. Evidence indicates that subclinical reactivation of VZV, and thus endogenous boosting, does in fact occur. DNA containing VZV genes appears in saliva of astronauts subjected to the severe stress of space travel without an accompanying episode of zoster (12) and has similarly been detected in the saliva of children subjected to the stress of hospitalization in an intensive care unit (13). VZV DNA also appears in the saliva of women in the last trimester of pregnancy, again without the manifestation of zoster (14). Endogenous boosting is a variable that was not entered into the computer models that have predicted that disastrous epidemics of zoster would follow the adoption of universal varicella vaccination.

Molecular differences between wild-type VZV and vOka, particularly in ORF62, have long been recognized (15). These are used as markers that enable infections with wild-type VZV and vOka to be distinguished. Although these markers are essential for the study of VZV epidemiology and pathogenesis, they have not been related to the attenuation of vOka (16); therefore, vOka has been used as an empirically documented safe and effective means of preventing disease with a molecular underpinning still to be defined. Given the recurrent fears that have dogged the use of varicella vaccine, prevented some countries from benefiting from it, and may yet emerge as undesired consequences of vaccination, knowledge of the molecular variations that contribute to the attenuation of vOka is important. Such knowledge should enhance understanding of the virulence of VZV. This knowledge should also help medicine cope not only with current fears about varicella vaccination but also with future problems due to VZV that may arise under circumstances that cannot now be predicted. The *tour de force* of application of modern methods of molecular and cell biology found in the work of Sadaoka et al. (3) has auspiciously begun to do just that.
